# The Effects of* Andrographis paniculata* on Platelet Activity in Healthy Thai Volunteers

**DOI:** 10.1155/2018/2458281

**Published:** 2018-08-06

**Authors:** Tichapa Sirikarin, Titchaporn Palo, Sirikul Chotewuttakorn, Weerawadee Chandranipapongse, Suveerawan Limsuvan, Pravit Akarasereenont

**Affiliations:** ^1^Department of Pharmacology, Faculty of Medicine, Siriraj Hospital, Mahidol University, Bangkok 10700, Thailand; ^2^Center of Applied Thai Traditional Medicine, Faculty of Medicine, Siriraj Hospital, Mahidol University, Bangkok 10700, Thailand

## Abstract

*Background. Andrographis paniculata* (AP) has been used in Thai traditional medicine to treat various infections, including the common cold and fever. Its bioactive compound, andrographolide, has shown antiplatelet activities in an* in vitro *study model. Since clinical studies of the effects of AP on the human platelet function have never been reported, we investigated its effect on platelet activity in ten healthy volunteers.* Methods*. Two grams of AP was taken 3 times within one day. The blood was withdrawn by venipuncture before and 2 and 24 hours after the AP administration to analyze the effects of AP on platelet aggregation, the expression of enzyme cyclooxygenase (COX) mRNA and protein, and TXB_2_, including P-selectin.* Result*. Even though there was no significant change in the studied parameters, this study exhibited patient-to-patient variability in platelet function. It was found that ADP-induced platelet aggregation tended to decrease after AP administration, while epinephrine-induced platelet aggregation in females tended to be higher than that in males for the entire study period. Moreover, COX-1 mRNA levels tended to decrease while P-selectin levels tended to rise after AP administration.* Conclusion*. These controversial results are possibly due to the multifactorial mechanisms of platelet aggregation as well as the multichemical composition of AP. Further study, probably at the molecular level, is needed to unveil the underlying mechanisms of action of AP.

## 1. Introduction

*Andrographis paniculata* (Burm. F.) Wall. ex. Nees has long been used to relieve sore throats, common cold, and fever in Asia and Scandinavia [1].* A. paniculata* is well known as Fa-Tha-Lai Chon in Thailand. It was included in the National List of Essential Medicines 2013 as a relief for the symptoms of the common cold and noninfectious diarrhea [2]. The active compounds of* A. paniculata* have exhibited therapeutic effects in many clinical and experimental studies [3]. Andrographolide is one of the major active compounds of* A. paniculata.*, playing an important role in the inflammatory process and cyclooxygenase (COX) enzyme expression [4, 5]. Moreover, some* in vitro* studies have shown that andrographolide inhibited platelet-activating-factor (PAF) induced platelet aggregation in humans [6] and thrombin-induced platelet aggregation in rats [7]. Platelets are essential for primary hemostasis, and they also have an important proinflammatory role. The activation of andrographolide may lead to the exposure of many adhesive molecules and inflammatory cytokines [8]. If* A. paniculata* exhibits antiplatelet effects, it may possibly be harmful to the physiological condition of hemostasis. However, there are still no clinical studies on the effects of* A. paniculata *on human platelet aggregation. This preliminary study is planned to evaluate the effects of* A. paniculata* on platelet activity, in particular, on platelet aggregation induced by a panel of agonists in healthy volunteers. The levels of COX production, TXB_2_ (a stable metabolite of TXA_2_), and P-selectin in aggregated platelets of the subjects taking* A. paniculata* were also evaluated.

## 2. Materials and Methods

### 2.1. Chemicals and Study Drugs

Vacutainer citrate tubes were purchased from BD (New Jersey, USA). High-quality grades of chemicals and reagents were purchased from Sigma (USA and Germany), Merck (Darmstadt, Germany), Bio-Rad (Germany), Cayman (USA), and GE Healthcare (USA).* A. paniculata* (AP) 500 mg capsules were obtained from the Manufacturing Unit of Herbal Medicines and Products, manufactured under GMP by Ayurved Thamrong School, Center of Applied Thai Traditional Medicine (CATTM), Faculty of Medicine, Siriraj Hospital, Mahidol University, Thailand. The powders contained 3% andrographolide (unpublished data from the unit of AP preparation). Therefore, one capsule of AP contained about 15 mg of andrographolide.

### 2.2. Subject Design

The study protocol and related materials were reviewed and approved by the Siriraj Institutional Review Board (SIRB) of the Faculty of Medicine, Siriraj Hospital, Mahidol University, Thailand. The approval (COA no Si 219/2014) included SIRB submission form, participant information sheet, informed consent form, case record form, advertisement for recruitment, and curriculum vitae. The 10 enrolled subjects comprised 5 males and 5 females, aged between 25 and 60 years. They were assessed as healthy, based on their medical history, a physical examination, clinical chemistry, and hematologic screening. A urine pregnancy test was performed on the female volunteers. All subjects were instructed to abstain from using any drugs that are known to affect platelet aggregation for at least 2 weeks before the study. They were also instructed to abstain from alcohol and cigarettes during the study. All participants provided verbal and written informed consent. In accordance with the National List Essential Medicines in Thailand [2] the participants received 3 × 2 g capsules of* A. paniculata* after meals within 1 day (a total of 6 g in the day). A pharmacokinetic study after the oral administration of 60 mg of andrographolide revealed that a maximum plasma level of approximately 1.9 *μ*M was achieved at 1.5-2 hours [9]. This level has been found to be enough to reveal some anti-PAF effects [6, 9]. To study the platelet aggregation, blood was therefore taken in the morning of the day of AP administration before the AP was dispensed (i.e., 0 hours) and then 2 and 24 hours after its administration.

### 2.3. Platelet Aggregation Assay

Citrated whole blood was centrifuged at 250 g for 10 min at room temperature to prepare platelet-rich plasma (PRP). Some PRP was further centrifuged at 4500 g for 2 min at room temperature to prepare platelet-free plasma (PFP) to set as a blank. Platelet aggregation was determined using light transmission (LTA) and Born's technique [10] in an aggregometer (AggRAM, Helena, USA). Since adjustment of PRP for platelet count does not provide any advantage [11] the extent of platelet aggregation using LTA was not significantly affected by platelet counts in the range 150–750 × 10^9^/L [12]; therefore time-consuming process of platelet count adjustment is not necessary unless outside of the range.

Epinephrine (Epi), adenosine diphosphate (ADP), and collagen (col) were used as a panel of platelet agonists. Briefly, PRP was incubated at 37°C for about 3 min before adding an agonist while stirring at 600 rpm. The reaction was allowed to proceed for 5 min. Then, the activated PRP was immediately placed in an icebox for further study of, for example, the expression of COX-1 mRNA including protein using platelet pellets and the levels of TXB_2_ and P-selectin using supernatant plasma. The maximum amplitude of the platelet aggregation was expressed as a percentage of the difference between the light transmissions of the aggregated PRP and PFP.

### 2.4. The Classification of Platelet Status

There is still no formal consensus on how to evaluate platelet function. Our laboratory therefore classified the function of the platelets into three patterns of aggregation (namely, disaggregation, or “dis”; normal aggregation, or “normal”; and hyperaggregation, or “hyper”), based on their reaction to various concentrations of epinephrine [13, 14]. The “disaggregation pattern” described the aggregation of platelets that were induced by 25 uM epinephrine and expressed as the primary phase of aggregation. By comparison, the “hyperaggregation pattern” was the name given to the aggregation of platelets that were induced by 1 uM epinephrine and expressed as the secondary phase of aggregation. Therefore, the activity of platelets to 1 *μ*M and 25 *μ*M epinephrine in a concentration-dependent manner was classified as a normal aggregation pattern.

### 2.5. Real-Time Quantitative PCR Analysis for COX-1 Expression

Protein suspensions of platelets were collected after the aggregated platelets were lysed with an ice-cold buffer and centrifuged at 10,000 x g for 10 min at 4°C. cDNA was synthesized using the SYBR PCR reagent kit (Applied Biosystems, Foster City, CA, USA), according to the manufacturer's protocol. Quantitative real-time PCR was performed by amplifying specific genes with FastStart SYBR Green Master (Roche Applied Science, Penzberg, Upper Bavaria, Germany).

The sequences of the primer sets were as follows:

  GAPDH forward primer: 5′-GACCACTTTGTCAAGCTCATTTCC-3′
  GAPDH reverse primer: 5′-TGAGGGTCTCTCTCTTCCTCTTGT-3′
  COX-1 forward primer: 5′-GACCCGCCTCATCCTCATAG-3′
  COX-1 reverse primer: 5′-CCACCGATCTTGAAGGAGTCA-3′
  COX-2 forward primer: 5′-CAAAAGCTGGGAAGCCTTCT-3′
  COX-2 reverse primer: 5′-CCATCCTTGAAAAGGCGCAG-3′.

The reactions were determined on the StepOnePlus Real-Time PCR system (Applied Biosystems). The cDNA samples, gene specific primers, were designed using vector NTI (Invitrogen) software. For data analysis, the mean cycle threshold (Ct) number from triplicate (n=10) was computed for each sample using the Sequence Detection Software (Applied Biosystems) with normalization to GAPDH as an internal control. PCRs products were determined on 1% agarose gels and stained with SYBR Green I Nucleic Acid Stain (Lonza, Rockland, USA).

### 2.6. Measurement of COX-1 Protein Using Western Blot Analysis

The total protein concentrations of the aggregated platelets were determined using the Bio-Rad protein assay reagent. An equal amount of total protein in each sample was loaded onto 10% polyacrylamide gel electrophoresis (SDS-PAGE). Immunoblotting using anti-COX-1 mouse monoclonal antibody was performed overnight at 4°C. The relative protein amount of gene expression was determined using Image Lab software (Bio-Rad).

### 2.7. Enzyme Immunoassay for TXB_2_ (a Stable Metabolite of TXA_2_) and P-Selectin Level

TXB_2_ (a stable metabolite of TXA_2_) and P-selectin (in supernatants of activated PRP) were measured using an enzyme immunoassay commercial kit, TXB_2_ EIA kit (GE Healthcare, Chicago, IL, USA), and P-selectin EIA kit (R&D Systems, Minneapolis, MN, USA), respectively. The supernatants of the activated PRP were processed for TXB_2_ and P-selectin according to the manufacturer's instructions.

### 2.8. Statistical Analysis

All reported values are the means of triplicate samples, and tests were repeated twice. The results were presented as mean ± standard deviation (SD). Data analysis was performed using GraphPad Prism version 5.03 and R software version 3.4.3. Two-way ANOVA was tested to find any differences in the parameters of the groups, followed by Bonferroni's post hoc test. P-values less than 0.05 were considered statistically significant.

## 3. Results

### 3.1. Subject Characteristic

The demographic characteristics of 10 healthy volunteers were shown in Table 1.

### 3.2. Effects of* A. paniculata* on Platelet Aggregation

**Figure 1:**
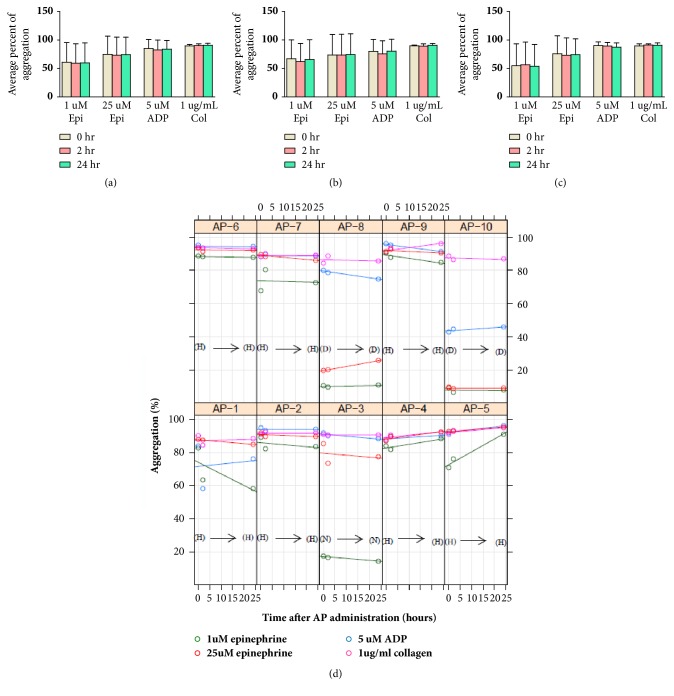
Platelet aggregation induced by a panel of agonists. Percentages of aggregation before (or 0 hours) and 2 and 24 hours after AP administration are shown as mean ± SD. (a) Total subjects; n=10. (b) Female subjects; n=5. (c) Male subjects; n=5. (d) Individual platelet aggregation of subjects; AP1-AP10 as percentage and patterns of aggregation; H=hyperaggregation, N=normal aggregation, and D=disaggregation.

At study entry, the platelet counts of PRP were in the range of 3.1-6.4x10^8^/mL. Aggregations of seven, one, and two subjects were classified to be of the hyper aggregation, normal aggregation, and disaggregation patterns, respectively. After AP administration, these patterns as well as the average of the percentage of aggregation induced by a panel agonist did not change for either females or males for the duration of the study, even though individual subjects showed minor changes in their platelet aggregations (Figure 1). After AP administration, platelet counts of PRP were in the range 3.5-5.7x10^8^ /mL and 2.8-5.0x10^8^/mL at 2 hours and 24 hours, respectively.

**Figure 2:**
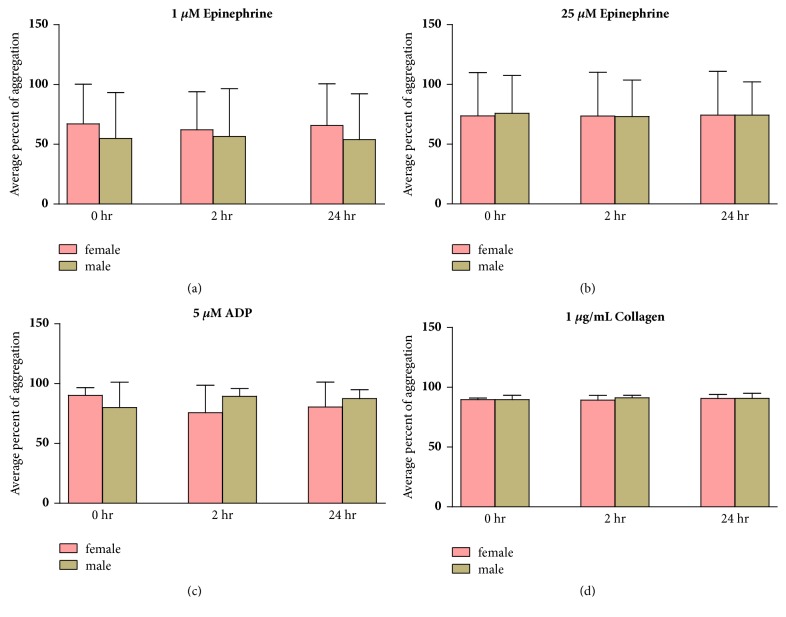
The effects of AP on platelet aggregation in females and males. Percentage of aggregation induced by a panel of agonists, (a) 1 *μ*M epinephrine, (b) 25 *μ*M epinephrine, (c) 5*μ*M ADP, and (d) 1 *μ*g/mL collagen, before (or 0 hours) and 2 and 24 hours after AP administration, are shown as mean ± SD.

However, there was a trend toward a higher platelet aggregation induced by 1 *μ*M epinephrine among females than males. Moreover, ADP-induced platelet aggregation showed a decreasing trend among females and an increasing trend among males after AP administration (Figures 2(a) and 2(c)).

### 3.3. Effects of* A. paniculata* on Expressions of COX-1 mRNA and Protein

**Figure 3:**
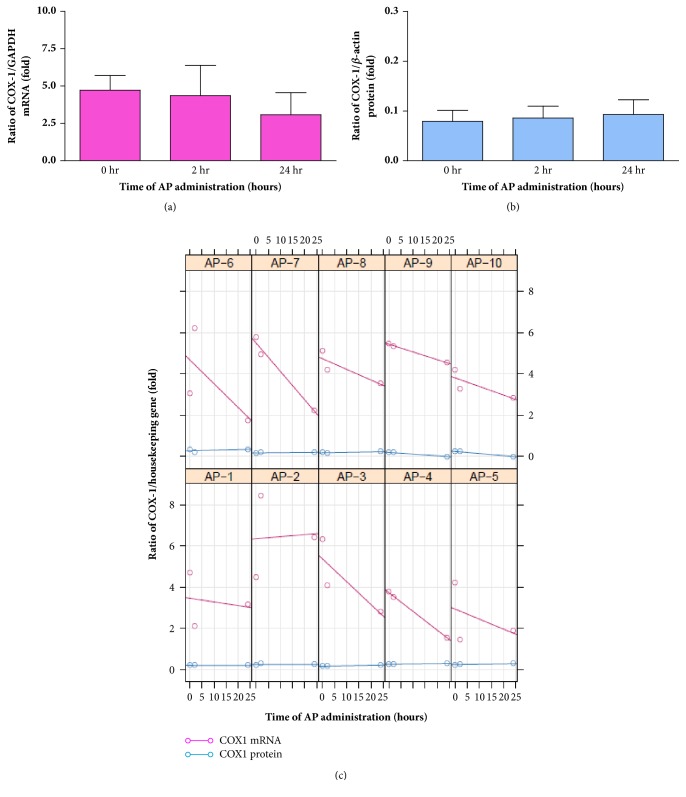
COX-1 mRNA and protein in platelets as the average fold of ratio/housekeeping gene. (a) COX-1 mRNA and (b) COX-1 protein, before (or 0 hours) and 2 and 24 hours after AP administration. (c) The individual expression of COX-1 mRNA and protein of subjects AP1-AP10.

Expressions of COX-1 mRNA and protein were detected in the platelets of all subjects. The average ratio/housekeeping gene of the COX-1 expression before AP administration was compared with that after AP administration, and they are shown as a percentage of control in Figure 3(b); the individual COX-1 expression ratio/housekeeping gene is shown in Figure 3(c). A trend toward decreased COX-1 mRNA was found after AP administration. When considering the individual results, most of the subjects in this study revealed a decreasing COX-1 mRNA; however, there was no significant change in the COX-1 protein.

### 3.4. Effects of* A. paniculata* on TXB_2_ and P-Selectin on Platelets

**Figure 4:**
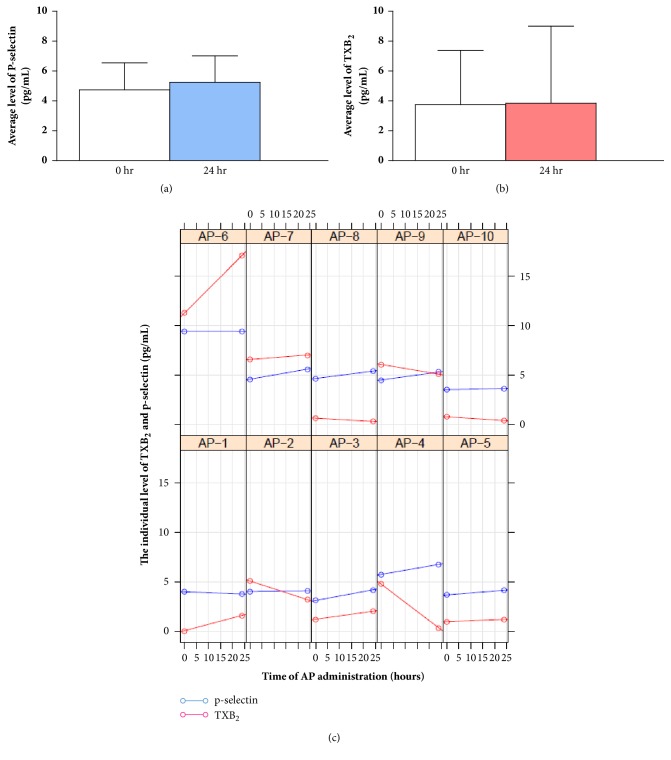
TXB_2_ and P-selectin in platelets before and after AP administration. (a) The average levels of TXB_2_. (b) The average levels of P-selectin. (c) The individual level of TXB_2_ and P-selectin in subjects AP1-AP10.

TXB_2_ and P-selectin in supernatant of PRP were investigated and are shown in Figures 4(a)–4(c). There was no significant difference in the level of TXB_2_, including P-selectin, before and after AP administration. However, when considering the individual results, most of the subjects in this study revealed an increasing level of P-selectin (Figure 4(c)).

## 4. Discussion

*Andrographis paniculata* (AP) is a Thai traditional medical herb that has been used to treat various infections, including the common cold and fever [2]. One of its major biological extract compounds, andrographolide, possibly contributes to those pharmacological actions. Andrographolide also possesses antiplatelet activity [6, 7, 15]. It inhibited PAF-induced human blood platelet aggregation [6] and thrombin-induced washed rat platelet aggregation [7] in the* in vitro* studies of Amroyan et al. and Thisoda et al., respectively. Moreover, another study by Lu et al. reported the potent antiplatelet activity of andrographolide; its activity was induced through the activation of the endothelial nitric oxide synthase- (eNOS-) NO-cyclic GMP pathway [15].

In our study, even though about 70% of the subjects exhibited platelet hyperaggregation for the duration of the study, some minor effects of AP on platelet aggregation were found. Those were that ADP induced platelet aggregation, showing a decreasing trend after AP administration (Figure 2(c)). This effect of AP might not be as clear as that shown in the studies by Lu et al., Amroyan et al., and Thisoda et al.

The difference is possibly due to the distinct pathway of platelet activation and the experimental models used in the study of platelet aggregation. Our study used the model of* ex vivo*, instead of* in vitro*, with a panel of different agonists (epinephrine, ADP, and collagen). Epinephrine is not considered to be a true mediator of platelet aggregation; however, it can potentiate the aggregatory response to ADP through coactivation of both the P2Y1 and P2Y12 receptors. ADP is a natural ligand for the P2Y1 and P2Y12 receptors, which play a crucial role in agonist-induced platelet aggregation. Collagen induces platelet-shape change and releases substances which then activate the platelets to aggregate, possibly involving the participation of the P2X1, P2Y1, and P2Y12 receptors, as well as thromboxane A2 (TXA_2_) formation [16, 17]. Taking into account the multifactorial mechanisms of platelet aggregation mentioned above and including the recently reported studies [18, 19] different platelet receptors are possibly involved in each pathway, with AP perhaps reacting differently with each receptor; this would lead to the variability in the platelet aggregation response.

The effects of AP on platelets, therefore, may vary depending on each individual person, as shown in Figure 1(d). However, the effects were probably not stable if a receptor was reversibly reacted. Moreover, substantial interindividual variations in drug responses among subjects may be governed by different drug metabolizing enzymes. Genetic variations may also be another important factor altering the effects of AP on platelets [20]. The other possible influencing factor is gender differences as there was a report that the platelets of females were more reactive than that of males; in addition, a gender difference in the response to epinephrine in platelet-rich plasma was found [21]. This agreed with our finding that the epinephrine-induced aggregations in females tended to be higher than that in males for the duration of the study (Figure 2(a)). However, there was also a study reporting a conflicting result in that sex difference did not affect platelet aggregation [22].

Although platelets are anucleate, they still contain COX-1, which generally provides physiological, housekeeping functions, such as the generation of proaggregatory TXA_2_ [23, 24]. This could be another factor contributing to patient-to-patient variability in platelet aggregation. We therefore also assessed the expression of COX-1 mRNA and protein, including the level of TXB_2_ and P-selectin in platelets. Even though there was no significant change in these parameters, trends toward decreasing COX-1 mRNA and increasing P-selectin levels were found after AP administration. These controversial results are possibly due to the multifactorial mechanisms of platelet aggregation as well as the multichemical composition of AP. Further studies by several scientific groups, probably at the molecular level, are needed to unveil the underlying mechanisms of AP action, especially on platelets.

Although our study is just a pilot screening for the existing effects of AP on platelet aggregation, the information obtained contributes more baseline data to the safety data sheet of AP. Since AP exhibits patient-to-patient variability in platelet aggregation, a surveillance protocol for the long-term use of AP should be performed, not only in patients with a bleeding tendency but also in those with thrombosis. Moreover, the information obtained should be used as guideline data for the planning of further studies.

## 5. Conclusions

**Figure 5:**
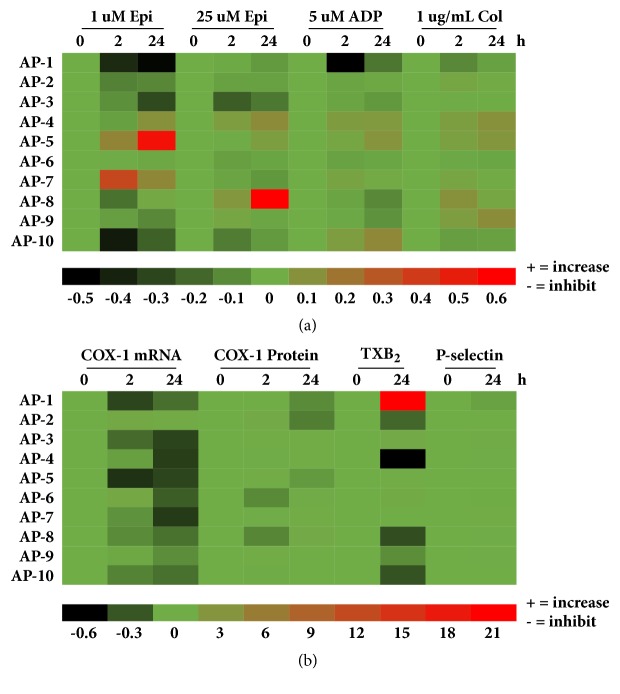
Heatmap analysis. Individual parameters after 2 and 24 hours of AP administration represent relative changes normalized by the 0-hour baseline of each subject AP1-AP10. (a) Parameters of platelet aggregation induced by a panel of agonists. (b) Parameters of the expression of COX-1, mRNA, and protein, including the levels of TXB_2_ and P-selectin, in resting platelets. Minus (-) and positive (+) value of color key indicate an increasing effect and an inhibiting effect of AP, respectively.

This study revealed preliminary data on the effects of* A. paniculata* on platelet aggregation induced by a panel of agonists (epinephrine, ADP, and collagen) in human volunteers. An individual variability in the platelet response to* A. paniculata* administration was found. Visualization of the results indicated a strong human volunteer variability, which led to difficulties in interpreting the effects of* A. paniculata*. However, it may also support the concept of personalized medicine based on platelet reactivity, as shown in Figure 5. Modulation of platelet reactivity by gender difference or products of the COX pathway, and P-selectin, should also be considered for* A. paniculata* usage, such as some pathological processes with a suspected bleeding tendency, including thrombosis. Further studies at the molecular level are necessary to reach conclusions.

## Figures and Tables

**Table 1:** Demographic characteristics and baseline laboratory values.

Baseline	Male (n = 5)	Female	Normal Range
		(n = 5)	
Age (years)	31.8 ± 6.2	41.2 ± 15.5	–
Body weight (kg)	71.1 ± 16.7	53.6 ± 6.5	–
High (cm)	170 ± 7.1	156.0 ± 6.5	–
Body mass index (kg/m^2^)	24.4 ± 4.0	22.1 ± 3.5	18–24
Hemoglobin (g/dL)	15.1 ± 1.6	13.3 ± 0.4	12.0–18.0
Hematocrit (%)	45.4 ± 3.8	40.3 ± 1.5	37.0–52.0
WBC (x10^3^/*μ*L)	6.9 ± 4.4	8.1 ± 5.3	4–11
Platelet count (x10^3^/*μ*L)	197.4 ± 83.6	262.4 ± 63.1	150–440
FBS (mg/dL)	89.0 ± 7.8	86.4 ± 5.5	74–100
Total cholesterol (mg/dL)	187.0 ± 59.9	230.4 ± 18.7	< 200
Creatinine (mg/dL)	1.0 ± 0.1	0.7 ± 0.01	0.51–0.95
AST (U/L)	23.3 ± 2.1	20.4 ± 4.2	0–32
ALT (U/L)	21.0 ± 4.6	19.0 ± 15	0–33

Data are presented as mean ± SD.

## Data Availability

The data used to support the findings of this study are available from the corresponding author upon request.

## References

[1] Jayakumar T., Hsieh C.-Y., Lee J.-J., Sheu J.-R. (2013). Experimental and Clinical Pharmacology of *Andrographis paniculata* and Its Major Bioactive Phytoconstituent Andrographolide. *Evidence-Based Complementary and Alternative Medicine*.

[2] Food and Drug Administration in Thailand (Thai FDA). Fah Talai Joan. National list of essential medicines, pp. 88-89, 2013

[3] Akbar S. (2011). *Andrographis paniculata*: a review of pharmacological activities and clinical effects. *Alternative Medicine Review*.

[4] Parichatikanond W., Suthisisang C., Dhepakson P., Herunsalee A. (2010). Study of anti-inflammatory activities of the pure compounds from *Andrographis paniculata* (burm.f.) Nees and their effects on gene expression. *International Immunopharmacology*.

[5] Chandrasekaran C. V., Gupta A., Agarwal A. (2010). Effect of an extract of Andrographis paniculata leaves on inflammatory and allergic mediators in vitro. *Journal of Ethnopharmacology*.

[6] Amroyan E., Gabrielian E., Panossian A., Wikman G., Wagner H. (1999). Inhibitory effect of andrographolide from *Andrographis paniculata* on PAF-induced platelet aggregation. *Phytomedicine*.

[7] Thisoda P., Rangkadilok N., Pholphana N., Worasuttayangkurn L., Ruchirawat S., Satayavivad J. (2006). Inhibitory effect of *Andrographis paniculata* extract and its active diterpenoids on platelet aggregation. *European Journal of Pharmacology*.

[8] Davì G., Patrono C. (2007). Platelet activation and atherothrombosis. *The New England Journal of Medicine*.

[9] Panossian A., Hovhannisyan A., Mamikonyan G. (2000). Pharmacokinetic and oral bioavailability of andrographolide from *Andrographis paniculata* fixed combination Kan Jang in rats and human. *Phytomedicine*.

[10] Born G. V. R. (1962). Aggregation of blood platelets by adenosine diphosphate and its reversal. *Nature*.

[11] Linnemann B., Schwonberg J., Mani H., Prochnow S., Lindhoff-Last E. (2008). Standardization of light transmittance aggregometry for monitoring antiplatelet therapy: An adjustment for platelet count is not necessary. *Journal of Thrombosis and Haemostasis*.

[12] Femia E. A., Scavone M., Lecchi A., Cattaneo M. (2013). Effect of platelet count on platelet aggregation measured with impedance aggregometry (Multiplate™ analyzer) and with light transmission aggregometry. *Journal of Thrombosis and Haemostasis*.

[13] Ketsa-Ard K., Juengchareon M., Poungvarin N., Jarerat S., Kittigul L. (1991). Clinical study on antithrombotic effects of ticlopidine in ischemic stroke. *Journal of the Medical Association of Thailand*.

[14] Gatina K., Barinov E., Sulaieva O. (2014). Sensitivity to Epinephrine Determines Platelet Hyperreactivity in Myocardial Infarction under Antiplatelet Therapy. *British Journal of medicine and Medical Research*.

[15] Lu W. J., Lin K. H., Hsu M. J., Chou D. S., Hsiao G., Sheu J. R. (2012). Suppression of NF-*κ*B signaling by andrographolide with a novel mechanism in human platelets: Regulatory roles of the p38 MAPK-hydroxyl radical-ERK2 cascade. *Biochemical Pharmacology*.

[16] Dorsam R. T., Kunapuli S. P. (2004). Central role of the P2Y12 receptor in platelet activation. *The Journal of Clinical Investigation*.

[17] Yun S.-H., Sim E.-H., Goh R.-Y., Park J.-I., Han J.-Y. (2016). Platelet Activation: The Mechanisms and Potential Biomarkers. *BioMed Research International*.

[18] Sunderland N., Skroblin P., Barwari T. (2017). MicroRNA Biomarkers and Platelet Reactivity: The Clot Thickens. *Circulation Research*.

[19] Zhang Y., Zhang J., Yan R. (2017). Receptor-interacting protein kinase 3 promotes platelet activation and thrombosis. *Proceedings of the National Acadamy of Sciences of the United States of America*.

[20] Oestreich J. H., Steinhubl S. R., Ferraris S. P., Loftin C. D., Akers W. S. (2014). Effect of genetic variation in P2Y12 on TRAP-stimulated platelet response in healthy subjects. *Journal of Thrombosis and Thrombolysis*.

[21] Miller C. H., Rice A. S., Garrett K., Stein S. F. (2014). Gender, race and diet affect platelet function tests in normal subjects, contributing to a high rate of abnormal results. *British Journal of Haematology*.

[22] Beyan C., Kaptan K., Ifran A., Savaşçi S., Öztürk Y., Ökmen B. (2006). Effect of sex difference on platelet aggregation using an optical method in healthy subjects. *Clinical & Laboratory Haematology*.

[23] Carey F., Menashi S., Crawford N. (1982). Localization of cyclo-oxygenase and thromboxane synthetase in human platelet intracellular membranes.. *Biochemical Journal*.

[24] Ebbeling L., Robertson C., McNicol A., Gerrard J. M. (1992). Rapid ultrastructural changes in the dense tubular system following platelet activation. *Blood*.

